# Compositional Dynamics: Defining the Fuzzy Cell

**DOI:** 10.1016/j.tcb.2016.08.012

**Published:** 2016-11

**Authors:** Georg Kustatscher, Juri Rappsilber

**Affiliations:** 1 Wellcome Trust Centre for Cell Biology, University of Edinburgh, Edinburgh EH9 3BF, UK; 2 Chair of Bioanalytics, Institute of Biotechnology, Technische Universität Berlin, 13355 Berlin, Germany

**Keywords:** organelle proteomics, machine learning, cellular map, gene ontology

## Abstract

Proteomic studies find many proteins in unexpected cellular locations. Can functional components of organelles be distinguished from biochemical artefacts or misguided cellular sorting? The clue might reside in compositional changes that follow biological challenges and that can be decoded by machine learning.

## The Fuzzy Cell

Textbook views of cellular components, from protein complexes to organelles, follow the paradigm ‘localization = function’. If a protein is found at a cellular location it also functions there. Consequently, the focus of organelle proteomics has been to get the localization right. For decades this was attempted by subcellular fractionation and by sorting out assumed contaminants. However, protein location may have other reasons than function: cellular components possess an intrinsic, compositional ‘fuzziness’.

An often overlooked feature of subcellular organization is that it results from affinities and equilibria, in other words is quantitative and not qualitative. Membranes act as barriers but also need to be permeable. The nuclear envelope, for example, is permeable to proteins smaller than ∼40 kDa. However, larger proteins might also make an uncontrolled entry into the nucleus, for example by having some affinity to the nuclear import machinery or at the end of mitosis, when the endoplasmic reticulum associates with the decondensing chromatin to reform the nuclear envelope. Proteins associated with chromosomes are included in this space, regardless of whether they are functional chromatin proteins or hitchhikers which decorate mitotic chromosomes as a result of their exposure to cytoplasm [1]. Possibly as a result of these processes, nonspecific association of proteins with genomic DNA has been observed also in interphase 2, 3.

The potential impact of dynamic equilibria is particularly obvious for the composition of non-membrane-enclosed compartments such as nuclear bodies and cytoplasmic granules. Proteins arrive there by diffusion and stay as a result of a preference for the environment of the respective compartment. However, it is highly unlikely that concentration gradients and local affinity will generate a binary sorting result, placing only proteins in those compartments that the cell needs to have there for functional reasons. For example, proteins appear not to exclusively localize to nucleoli, despite their enrichment there [4].

The cell does tolerate sorting noise. Possibly it is an essential part of evolution, allowing proteins to acquire a local function under some selection pressure. Proteins can acquire new subcellular localizations during evolution, as seen for duplicated gene pairs in yeast, which frequently possess functions in different organelles [5]. Proteins can also occupy multiple subcellular compartments as a result of their biosynthesis, transport, maturation, storage, or regulation. These processes are necessary for proteins to arrive in mature form at the location where they function. Consequently, transitory locations are ‘true’ locations of these proteins but not the sites of their function. Finally, multifunctional proteins exist that localize and function in multiple organelles, such as the mitochondrial prohibitins that ‘moonlight’ as nuclear transcription factors.

In the most recent draft of an organellar map of proteins it was noted that almost half of the observed proteins could not be assigned to discrete cellular locations [6]. Therefore, fuzziness appears to be a widespread phenomenon. If these proteins are to be placed onto a cellular map a different approach is needed. We propose a new concept to describe cellular organization, which combines indicators of protein function with localization data in a probabilistic framework.

## A Potential Solution: Adding Function to Localization

Methods will have to be developed that can distinguish between proteins that function at a location and those that are present owing to biological leakiness or imperfections in purification. This requires spatial data (colocalization or co-fractionation) to be combined with sources of protein function. One potential way of achieving this is to use machine-learning algorithms to integrate a variety of data sources that include this information (Box 1).

**Figure 1:**
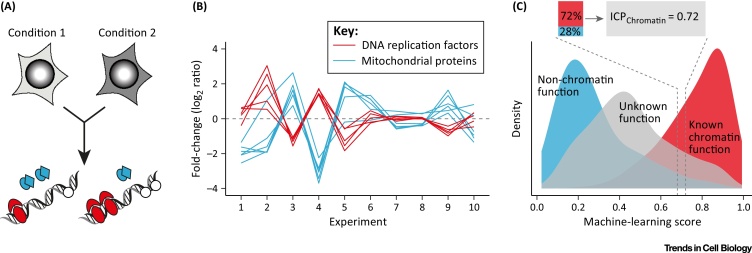
Functional Organelle Components Can Be Identified Through Covariation. In this example, chromatin was enriched from cells grown under different conditions, for example following drug treatments [3]. (A) Chromatin fractions contain both *bona fide* chromatin proteins (red: e.g., DNA replication factors) and proteins which are unlikely to have chromatin-based functions (blue: e.g., mitochondrial proteins) and uncharacterized factors (white). (B) Proteins with similar functions tend to show coordinated changes between different experiments. Such covariation patterns can be used by machine-learning algorithms to identify functional components of an organelle [3]. (C) Proteins can be assigned to an organelle using integrated compartment probability (ICP). The machine-learning score ranks proteins according to how similar their behavior is to known functional components of the organelle. To turn the score into a probability, the score distribution of known functional components is put in relation to that of proteins that definitely do not function in the organelle. In this example, the distribution of known chromatin factors is strongly skewed towards higher scores, whereas proteins without chromatin-based functions, such as cytoplasmic, metabolic enzymes, tend to score low. The proportions of the two distributions correspond to the probability with which any uncharacterized protein (grey) in a given score window will have a function in chromatin. The DNA replication factors shown in (B) are SSRP1, MCM7, RFC1, RPA1, and REPIN1. The mitochondrial proteins are ATP5A1, TOMM70A, FH, LONP1, PDHB, and HADHA.

Using this approach, many studies have built on mRNA or protein covariation across multiple biological experiments as the source of functional data. To generate a compendium of mitochondrial proteins, mitochondrial fractionation proteomics has been combined via naïve Bayes machine learning with additional data, including mRNA coexpression and sequence features such as the presence of mitochondrial target peptides or characteristic protein domains [7]. For mitotic chromosomes, a combination of proteomic data and domain annotation was used to segregate putatively functional components from hitchhikers [1]. This led to the observation that function at a subcellular location can also be inferred from proteomics data alone. This follows a two-step procedure: first, proteins are quantified across multiple biochemical isolations of a cellular structure, obtained from differently perturbed cells as starting material 3, 8. Second, one determines the covariation of all identified proteins with known functional components of that organelle (Figure 1). Proteins with similar functions tend to behave more similarly to each other than to unrelated proteins across different biological conditions, for example in response to drug treatments or cell differentiation. The ‘behavior similarity’ or covariation can be measured using multi-classifier combinatorial proteomics (MCCP) [1], which is based on another machine-learning approach, random forests. So far, both chromatin components 1, 3 and mitochondrial proteins [8] can be determined on the basis of their covariation, suggesting this could be a general method to determine functional organelle composition and an alternative to approaches based on co-fractionation. Indeed, covariation was better suited to distinguish functional from non-relevant chromatin-bound proteins than classical, purification-based approaches [3]. Protein covariation can also inform on organelle composition for organelles that contaminate the biochemical purification of another organelle [8]. In principle, the more different biological conditions that are tested for the composition of an organelle, the better one can capture its constitutive, functional components. Importantly, instead of choosing an arbitrary cutoff to separate genuine organelle components from contaminants, machine-learning scores could be turned into a probabilistic version of gene ontology that fuses functional and localization considerations. A first example could be seen in interphase chromatin probability (ICP), possibly rephrasing ICPs as ‘integrated compartment probabilities’ [3] (Figure 1C). ICPs can be generated relatively easily for cellular structures of interest, provided that training sets and proteomics data for the species are available. The outcome is a list of all proteins detected in the analysis together with their probability of being a functional component of that organelle. An ICP of 0.8 predicts that 8 of 10 uncharacterized proteins with this value have a functional link to the organelle. One limitation of this approach is that it only works for organelles with sufficiently well-characterized components, although training sets do not need to be large because MCCP has been applied to protein complexes 9, 10.

## Application of Compartment Probabilities in Targeted Studies

ICPs are being applied. Proteomics experiments typically distinguish between relevant proteins and background through quantitative comparison. For example, DNA replication factors could be identified because they are enriched on replicating chromatin over mature chromatin. However, because these two chromatin states differ in their protein composition they also attract different background proteins [11]. Consequently, not all proteins that differ significantly between these two states are related to DNA replication. More than half of 1000 well-characterized proteins enriched on replicating chromatin were classified as biochemical contaminants because they were known to function elsewhere in the cell. This made it difficult to select candidates for novel DNA replication factors among the 300 co-enriched uncharacterized proteins. Filtering the dataset for proteins with high chromatin ICPs removed 90% of the contaminants, while retaining 90% of the known replication factors, and pinpointed 93 uncharacterized proteins as promising candidates for follow-up studies. Experimental validation for seven uncharacterized proteins enriched on replicating chromatin confirmed that three with high ICPs were indeed chromatin-based, and four with low ICPs were indeed background [11]. Likewise, ICPs guided the analysis of Cdk-dependent changes in S-phase chromatin. Of 114 proteins whose chromatin association was significantly and reproducibly dependent on Cdk activity, more than half were considered to be contaminants and 90% of these could be removed by ICP-based filtering [3]. Interestingly, the concept of protein covariation can also inform on the inner organization of organelles. For example, the relationship between protein complexes and novel complex components could be studied in the context of intact mitotic chromosomes 1, 10.

## Concluding Remarks and Future Directions

Not every cellular localization of every protein has a functional consequence, and we need tools that will allow us to disentangle those that do from those that do not. This will enhance our ability to study cellular processes, and will increase our appreciation and understanding of the cell at a systems level. As more evidence for proteins existing in multiple cellular components accumulates, purely qualitative annotations will become more limited. Such annotation efforts have been essential for biological research in the past, but categorical annotation, without information on functionality for many proteins, risks becoming meaningless. While we currently have only acquired probabilities for chromatin- and for mitochondria-based function, future experiments will reveal the probability with which these and other proteins function in other organelles. Over time, it could lead to a quantitative, big-data-driven map of the cell, describing where each protein is present, and more importantly, where their functions are.

## Box 1 Machine-Learning Tools in Organelle Proteomics

Machine learning is concerned with the implementation of computer software that can learn autonomously [12]. In a typical scenario of organelle proteomics, organelle composition is learned from quantitative proteomics data, often including categorical data (e.g., the presence of a localization signal). In supervised machine learning, a positive training set (known components of an organelle) and a negative training set (proteins known not to be part of that organelle) are used to train the algorithm to identify additional unknown components. Algorithms used include random forests to define components of mitotic chromosomes [1] and interphase chromatin [3], a naïve Bayes framework for mitochondria [7], as well as k-nearest neighbors and support vector machines for a combination of organelles 6, 13. An alternative approach is unsupervised machine learning. These algorithms do not require training sets and instead divide data into clusters or groups of proteins. Unfortunately, these clusters do not necessarily coincide with what one is looking for. Principal component analysis (PCA) has been used as the main algorithm for this approach, for example to define clathrin-coated vesicles [14] and multiple membrane-bound organelles in a single experiment [15].
